# On the origin of emission and thermal quenching of SRSO:Er^3+^ films grown by ECR-PECVD

**DOI:** 10.1186/1556-276X-8-98

**Published:** 2013-02-22

**Authors:** Artur Podhorodecki, Grzegorz Zatryb, Lukasz W Golacki, Jan Misiewicz, Jacek Wojcik, Peter Mascher

**Affiliations:** 1 Institute of Physics, Wroclaw University of Technology, Wybrzeze Wyspianskiego 27, Wroclaw 50-370, Poland; 2 Department of Engineering Physics and Centre for Emerging Device Technologies, McMaster University, Hamilton, ON, L8S 4L7, Canada

**Keywords:** Silicon, Nanocrystals, Erbium, Emission, Quenching, Temperature, Photoluminescence, 78.67.Bf, 73.63.Bd, 78.40.Fy, 78.55.Ap

## Abstract

Silicon nanocrystals embedded in a silicon-rich silicon oxide matrix doped with Er^3+^ ions have been fabricated by electron cyclotron resonance plasma-enhanced chemical vapor deposition. Indirect excitation of erbium photoluminescence via silicon nanocrystals has been investigated. Temperature quenching of the photoluminescence originating from the silicon nanocrystals and the erbium ions has been observed. Activation energies of the thermally activated quenching process were estimated for different excitation wavelengths. The temperature quenching mechanism of the emission is discussed. Also, the origin of visible emission and kinetic properties of Er-related emission have been discussed in details.

## Background

Rare-earth-doped materials have been investigated for a number of years in order to develop practical light sources for photonic applications. A silicon-rich silicon oxide (SRSO) matrix seems to be very promising as an efficient photosensitizer for different rare-earth (RE) ions such as: Nd^3+^[1,2], Tb^3+^[3], or Er^3+^[4,5]. Among these ions, the Er^3+^ ion is well known as an alternative to epitaxially grown light sources emitting in the third telecommunication window [6,7]. One of the advantages of a SRSO matrix as a host for RE ions is the formation of Si nanocrystals (Si-NCs) within the matrix which could participate in indirect excitation of Er^3+^ ions via an energy transfer process. Additionally, these clusters can improve the film’s conductivity, which in practice can be an even more important benefit. The advantage of using Si-NCs comes from their high absorption cross section (σ_abs_) as compared to very low ones for most of the RE ions. For example, for erbium in SiO_2_, the experimentally determined value of σ_abs_ is 8 × 10^-21^ cm^2^[8], while for Si-NCs at 488 nm, this value is equal to 10^-16^ cm^2^[9]. Moreover, Franzo et al. [10] and Gourbilleau et al*.*[11] reported already that amorphous Si nanoclusters (*a*Si-NCs) can be sufficient and even better sensitizers than Si-NCs, enhancing the optical activity of Er^3+^ ions. Thus, enriching SiO_2_ with Si nanocrystals or amorphous nanoclusters should significantly increase Er^3+^ emission due to their indirect excitation. However, to date, achieving gain from this material has proven to be a notoriously difficult task. This is, in part, due to the low excitable Er^3+^ fraction sensitized through the Si-NCs (0.5% to 3% [12,13]) and the low number of excitable Er^3+^ ions per nanocrystal (1 to 2 [14,15] or 20 [12]), which affects the maximum gain that can be achieved in a Si-sensitized gain medium. It is believed that the low number of optically active Er^3+^ ions coupled to Si-NCs is due to processes like fast Auger back-transfer from excited Er^3+^ ions to excitons in Si-NCs, excited-state absorption, or Er^3+^ pair-induced quenching. Nevertheless, experimental data strengthening or excluding any of these explanations is still limited. One of the exceptions is recent work of Navarro-Urrios et al. [16] who have shown that none of these processes are responsible for the low fraction of Er^3+^ coupled to Si-NCs, and only the short range of interaction between Si-NCs and Er^3+^ (0.5 nm) is the main limitation to achieving a high fraction of ions coupled to Si-NCs. As a consequence, it has been shown that the amount of excitable Er^3+^ depends strongly on the Si-NC density as only those Er^3+^ ions in close proximity to the Si-NCs are being excited [17]. Therefore, it is believed that the main limitation on obtaining gain in such a system is the low density of sensitizers, the short range of the Si-NCs and Er^3+^ interaction [13], and low solubility of Er^3+^ ions in SRSO matrix.

However, in addition to weak Si-NCs-Er^3+^ coupling, reduction in Er^3+^ emission efficiency can also be due to many quenching processes accompanying the excitation and recombination of Er^3+^ ions. Such processes still have not been widely investigated. Furthermore, even today, the detailed excitation mechanism of Er^3+^ ions in SRSO is still not well understood. Investigations of time-resolved photoluminescence of Er^3+^ ions in SRSO reveal two major excitation mechanisms leading to 1.5-μm emission, distinguishable by their dynamics: a fast relaxation within the Si-NCs and energy transfer to ions (<100 ns), taking Er^3+^ ions directly to the first excited state, and a slow relaxation and energy transfer, exciting Er^3+^ ions to higher states. In both cases, however, the emission decay should be slowed down due to slow radiative relaxation from ^4^*I*_13*/*2_ to ^4^*I*_15*/*2_ on a millisecond-microsecond time scale [18-20]. The fast energy transfer has already been related to Auger-type excitation of Er^3+^ ions directly from the Si-NCs to ^4^*I*_13*/*2_ level of Er^3+^ ions. In this case, excited ions should be inside the core of Si-NCs or at their surface due to the short range of Auger-type interactions. This mechanism can also be discussed since to obtain a high efficiency of Auger recombination within the Si-NCs, the energy levels of Si-NCs should be well separated from each other to minimize thermal relaxation which strongly reduces the Auger-type relaxation. It has been shown, however, theoretically that for Si-NCs, especially when surface/matrix interface is included into the calculations, the energy spectrum of Si-NCs is almost continuous above the main absorption edge [21,22]. Besides, it has been shown recently that in the spectral range of Er^3+^ emission, another emission with nanosecond decay appears which, however, cannot be related to Er^3+^ ions. This emission can be assigned more likely to defect states in the SRSO film. Thus, many open questions regarding the origin of the fast process still remain.

It is widely believed that the slow process is due to dipole-dipole energy transfer either from the exciton confined inside the Si-NCs or localized at their surface states. In this case, the transfer can occur efficiently (with a rate of 10^9^ s^-1^) to the ions located even 6 to 7 nm from the Si-NCs, as has been shown by Choy et al. [23]. On the contrary, other authors have proposed that the optimal distance between Si-NCs and Er^3+^ ions is on the order of 0.5 nm only [24,25]. With such a short interaction distance, the question regarding the nature of energy transfer and validity of dipole-dipole interaction only became important. Moreover, in case of slow energy transfer, the intermediate defect states in the SRSO matrix became important and can also participate in Er^3+^ excitation allowing exciton migration before the exciton transfers its energy to Er^3+^ ions. This should also increase the distance of Si-NC-Er^3+^ interaction. In view of the above discussion, understanding of the origin of visible emission and temperature quenching of the emission of Er^3+^ becomes important to understand the efficiency of coupling between Er^3+^ and Si-NCs as well as the energy transfer between them.

There are several processes which might be responsible for temperature quenching of the photoluminescence (PL) in Si-NCs, such as (a) carriers’ resonant/non-resonant tunneling out of Si-NCs to sites where the non-radiative recombination occurs [26], (b) thermal activation of carriers over the potential barrier Si/SiO_2_ (3.4 eV) [27], and (c) simply non-radiative band-to-band transition. Other potential mechanisms, such as exciton dissociation (approximately 14 meV for bulk Si [28]) should rather be excluded from consideration in the case of Si-NCs since within the Si-NCs, there are no excitonic levels other than the ones related to Si-NCs itself, where the Columbic interaction has been included in self-consistent calculations. Thus, there are no additional levels to which the exciton could dissociate as in the case of bulk material. The only quenching energy which could be associated with *exciton dissociation* is one which moves one of the carriers to defect levels at the surface of Si-NCs over the potential barrier (process a or b). The only temperature-dependent emission-quenching mechanisms related to the *excitonic nature* of carriers confined within the Si-NCs can be due to different spin selection rules for different energy levels, which give the dark and bright states, which can be split in Si-NCs even with 20 meV [29].

In the case of erbium ions, PL quenching can be related to back-transfer mechanisms [30], which should be, however, very inefficient in Si-NCs because of the large difference in Er^3+^ emission energy and the absorption edge of Si-NCs [31]. Another common mechanism responsible for the quenching of PL originating from Er^3+^ is Auger recombination between excited Er^3+^ and excess electrons bound to a surface/defect state at Si-NCs [32]. Finally, Er^3+^ can transfer energy due to dipole-dipole interactions to other ions or to defect states which play the role of quenching centers. In order to be temperature-dependent, all these quenching processes should be phonon assisted.

In view of the above discussion, it can be seen that even if work on SRSO: Er^3+^-based LEDs is already advanced [7] from the fundamental point of view, there are many uncertainties and contradicting results in the literature. We believe that one of the main reasons is simplification of the interpretation of the obtained emission signal as related to Si-NCs only and the unappreciated role of the complex nature of the SRSO film where defects and both *a*Si-NCs and Si-NCs can be optically active simultaneously in the same spectral range. Moreover, in many cases, the 488-nm line is used for SRSO:Er^3+^ excitation, where this wavelength overlaps with one of the optical transitions of Er^3+^ ions and can bring about interpretation of obtained data.

In this work, we present new data regarding excitation, recombination, and thermal quenching of Er^3+^-related emission. As the absorption cross sections of Si-NCs and Er^3+^ ions are different by orders of magnitude, the excitation of Er^3+^ via Si-NCs at low excitation power should dominate over their direct excitation. Thus, as an additional aim of this work, we examine the optical properties of SRSO:Er^3+^ at an excitation truly resonant with 4*f-4f* energy levels (980 nm), at indirect excitation (266 nm), and at 488-nm excitation wavelength, the non-resonant nature of which is questionable.

## Methods

The Er-doped SRSO film was grown on a Si substrate by electron cyclotron resonance plasma-enhanced chemical vapor deposition (ECR-PECVD) using SiH_4_ and O_2_ source gases diluted in Ar to form the SRSO matrix. Er(TMHD)_3_ was employed as the rare-earth precursor to achieve high concentrations of Er doping. The film was annealed in a quartz tube furnace under flowing ultrahigh-purity N_2_ for 1 h. The annealing temperature was 1,100°C. As we have shown in many previous papers, in our deposition system, this temperature is sufficient to obtain silicon nanocrystals of a few nanometers in size, both in the absence of erbium doping [33] and in the case of doping with erbium and different lanthanides [33,34]. The deposition system has been described in detail elsewhere [33]. The composition of the film (39 and 37 at.% of Si and 0.45 at.% of Er) was measured by Rutherford backscattering spectrometry. The film thickness estimated from ellipsometry experiments was 200 nm for both samples.

The room-temperature photoluminescence excitation (PLE) of the erbium ions in the near-infrared (NIR) was measured using an InGaAs pin photodiode. As an excitation source, a 450-W Xe arc lamp connected to a Triax 180 monochromator (Jobin-Yvon, Kyoto, Japan) was used. PL as a function of temperature was excited using a 488-nm Ar^+^ CW laser (Melles Griot, Albuquerque, NW, USA), 266-nm (Elforlight, Daventry, UK) and 980-nm (Opolette™, Opotek Inc., Carlsbad, CA, USA) pulse lasers. An HR4000 spectrometer (Ocean Optics, Dunedin, FL, USA) and InGaAs CCD linear detector (Symphony^®^ I line, Horiba Jobin-Yvon) were used as detection systems for measurements in the visible (VIS) and NIR spectral range, respectively. The PL decay was measured using pulsed laser coupled to a gated detection system (QuantaMaster from Photon Technology International, London, Canada).

## Results and discussion

**Figure 1:**
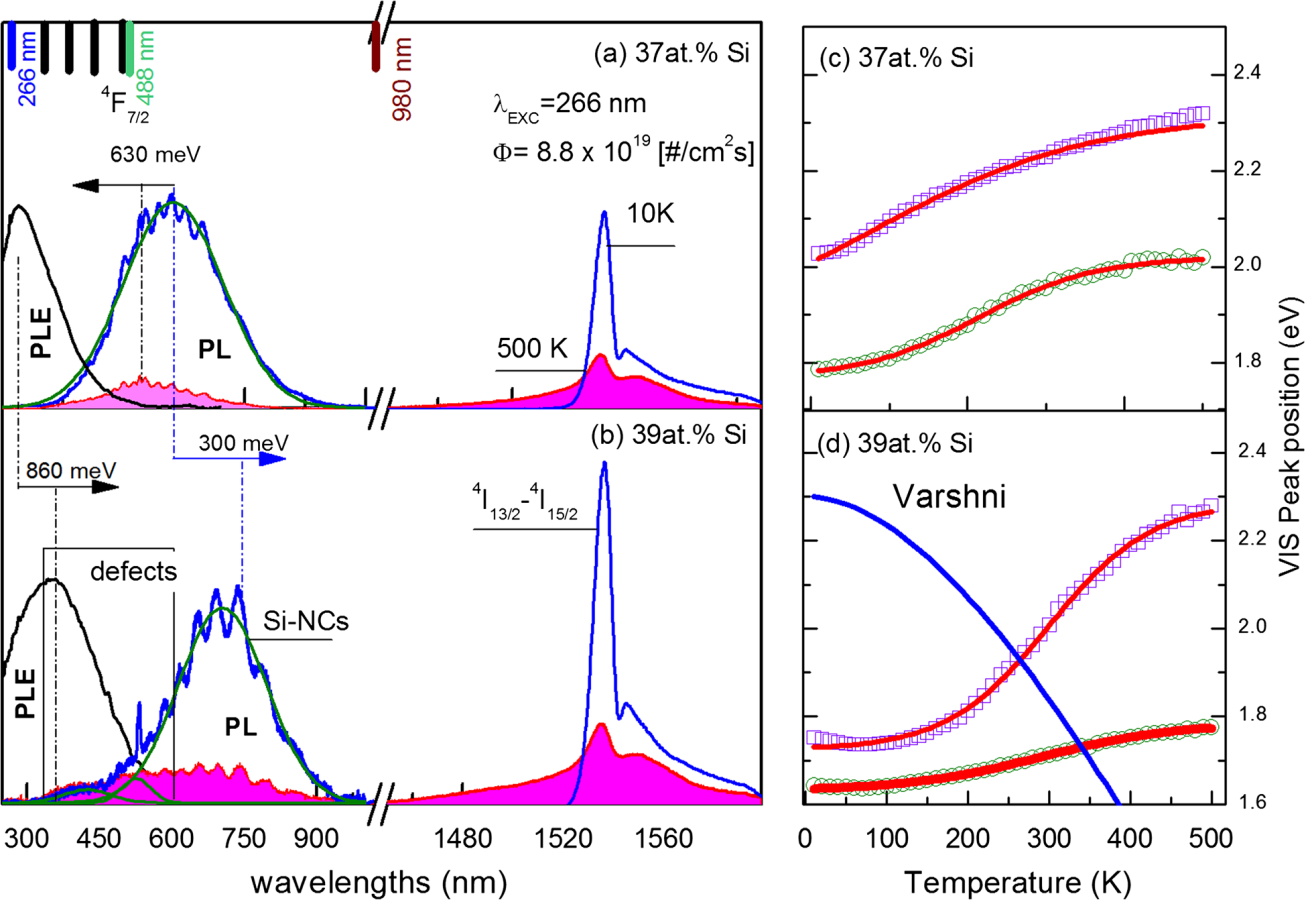
**Results of photoluminescence measurements. **PL spectra of Si-NCs (VIS) doped with Er^3+^ (NIR) measured at 10 and 300 K at 488-nm excitation together with normalized PLE spectra detected at 0.81 eV for two Si concentrations: (**a**) 37 at.% and (**b**) 39 at.% of Si. The normalization was done for both spectra separately. Emission peak positions as function of temperature for two excitation wavelengths, 266 (squares) and 488 nm (circles), for two different Si concentrations, (**c**) 37 at.% and (**d**) 39 at.%, together with theoretically predicted Varshni formula. For the Varshni formula, Si bandgap at 0 K has been set as 2.3 eV for better data presentation.

Figure 1a shows the PL spectra of SRSO films doped with Er^3+^ ions measured at 500 and 10 K for samples with two Si atomic concentrations: 37 and 39 at.%. Two main emission bands at 1.6 and 0.81 eV have been observed. The first band at 0.81 eV is assigned to a radiative intra-4*f* shell transition of Er^3+^ ions (^4^*I*_13/2_ → ^4^*I*_15/2_). The shape of this band at a high temperature can be modeled with a Boltzmann distribution for thermal populations of the crystal-field split manifold of ^4^*I*_13/2_ and ^4^*I*_15/2_ sublevels that have a total of 56 possible transitions between them, giving significant contribution to the observed broadening of this band.

The second band at 1.6 eV can be assigned to the recombination of excitons localized in the SRSO matrix. Moreover, from Figure 1a, it can be seen that all VIS emission bands have a complex structure. This is due to interference effects caused by the refractive index contrast between SRSO and the Si substrate [35]. These interferences will modify the shape of the emission spectra in the entire VIS spectral range. However, Er^3+^ emission is not affected by this effect.

Additionally, Figure 1a shows the PLE spectra measured for Er^3+^ at room temperature at 0.81 eV in a broad UV-VIS excitation band energy range. The obtained PLE spectra are also very similar to those obtained by us for undoped SRSO samples [36,37]. The appearance of strong Er^3+^ emission at excitation wavelengths far from resonance with erbium energy levels clearly indicates that we are dealing here with an efficient excitation transfer from the levels responsible for VIS emission (i.e., *a*Si-NCs, Si-NCs, or defects) to erbium ions. The main argument behind the conclusion that defect states can be excluded in this case is the Si-concentration-dependent position of the excitation spectra for Er^3+^ ions and VIS emission bands. It can be seen that when the Si content increases, the edge of excitation as well as emission bands shifts towards lower energies due to reduction of quantum confinement. This suggests that the observed VIS emission can be related either to *a*Si-NCs or to Si-NCs. Moreover, the position of these excitation bands at 4.3 and 3.4 eV for 37 and 39 at.% of Si, respectively, seems to be different than energies typically obtained for excitation bands of defects in SiO_2_ films: ‘non-bridging oxygen hole center’ at 4.8 and 5.8 eV [38], *E*’ center at 5.4 to 6.2 eV [39], or ‘oxygen-deficient center’ (ODC) at 7.6, 6.9, and 5.0 eV [40].

Another important conclusion from Figure 1a is that the emission band in the VIS spectral range cannot be assigned to Si-NCs or *a*Si-NCs only, but some contribution from defect states can also be clearly observed, especially for the sample with 39 at.% where weak emission bands at around 450 nm can be observed. These defect states are most probably due to ODC in the SiO_2_ matrix [41] or self-trapped excitons (STE) [42]. It has been shown that emission from ODC at this energy is characterized by a long emission decay time of 10 ms [40]. On the other hand, the emission decay time of STE should rather be in the nanosecond range. However, the nature of STE in SiO_2_ is not clear at the moment. Nevertheless, we believe that emission at 1.6 eV originates mainly from *a*Si-NCs where the recombination is due to transitions between the tails of local density of states (LDOS) related to *a*Si-NCs rather than to the band-to-band excitonic transitions like in Si-NCs. One of the arguments strengthening our hypothesis can be seen in Figure 1c,d where the VIS emission peak position has been monitored with temperature ranging from 10 to 500 K for two excitation wavelengths. The PL peak position shows abnormal blueshift with increasing temperature. Usually, the PL peak position for unalloyed semiconductors shows a redshift with increasing temperature in accordance with Varshni’s formula [43] shown also in Figure 1b with parameters typical for bulk Si. The temperature dependence of the PL peak position shown in Figure 1d is rather similar to the S-shaped phenomenon observed due to localized states caused by potential fluctuations in semiconducting alloys [44]. This should be a similar case for amorphous clusters. This is mainly because the tail states (*N*_tail_) of *a*Si-NCs can be approximated as an exponential distribution [45],

(1) $$N_{\text{tail}}\left(E\right)=N_{\mathrm{TC}}\cdot \text{exp}\left(\frac{E-E_{m}}{kT_{t}}\right).$$

Based on Equation 1, the carrier density trapped at localized tail states (*n*_tail_) can be estimated using the Fermi-Dirac statistics,

(2) $$n_{\text{tail}}\left(E,T\right)=\int_{E_{F0}}^{E_{m}}N_{\text{tail}}\left(E\right)\cdot f\left(E\right)\mathrm{dE}$$

where *f*(*E*) is the Fermi probability function defined as *f*(*E*) = [1 + exp(*E* - *E*_*F*_/*kT*)]^-1^, where *k* is Boltzmann’s constant and *T* is the ambient temperature. Thus, at a low temperature, carriers relax to the lowest levels within the tails of LDOS. However, when the temperature increases, carriers move to higher lying levels and recombine at higher energies. Moreover, due to the increased role of non-radiative channels at a high temperature, the emission decay time is reduced, and thus, carriers can recombine from higher levels, also moving the emission band towards higher energies. Thus, the observed emission band at 1.6 eV can be related mainly to *a*Si-NCs. However, we cannot exclude additional contributions to the observed emission from Si-NCs.

From Figure 1, we can clearly see the redshift of the total VIS emission with increasing Si content. Based on the above results, the observed shift can be explained as due to changes in *a*Si-NC sizes (redshift due to quantum confinement effect), changes in number of defect states making contributions to tails of LDOS (blue- or redshift), relative contribution of emission bands from matrix-related defect states, or Si-NC- and *a*Si-NC-related emission. Moreover, increasing strain at the Si-NCs/SiO_2_ interface with Si atomic percent should also be included as it has been shown by us recently elsewhere [46]. This can introduce defect states and can also influence the position of the energy levels related to Si-NCs inducing both red- and blueshift.

**Figure 2:**
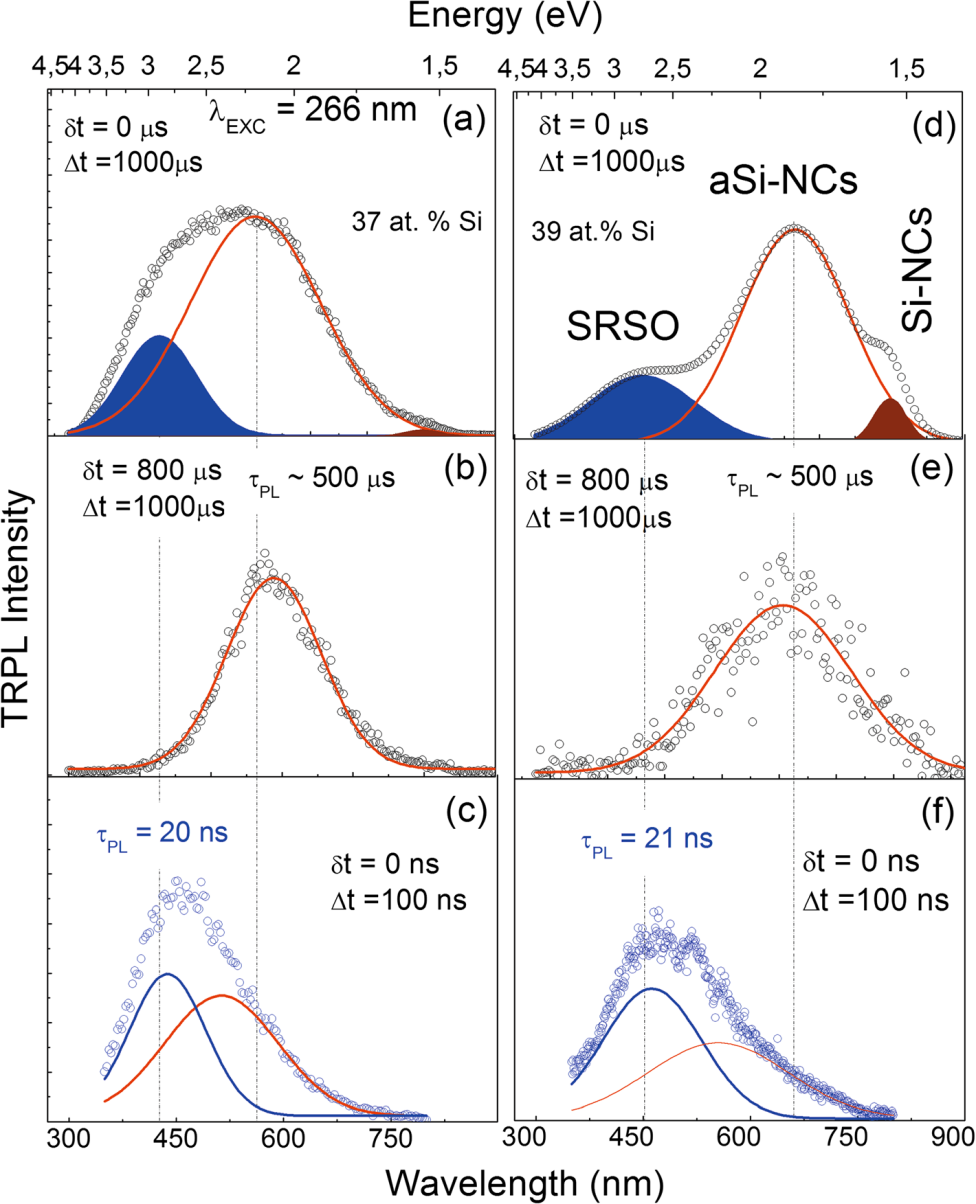
**Time-resolved PL spectra. **SRSO:Er^3+ ^samples obtained at 266-nm excitation for (**a**, **b**, **c**) 37% and (**d**, **e**, **f**) 39% of Si. Δ*t*, integrating time; Δ*t*, delay time.

To analyze in detail the origin of the observed VIS emission bands, time-resolved PL spectra (TRPL) have been measured for two samples at 266-nm excitation wavelength. Obtained results are shown in Figure 2. Figure 2a,d shows emission spectra obtained just after the excitation with a laser pulse of less than 2 ns wherein the signal was collected during 1,000 μs. This condition should best reflect the emission signal obtained at the CW excitation shown in Figure 1. As it has been discussed already, the observed emission is composed of at least three independent emission bands overlapping each other spectrally. When the delay between the pulse and detection is set to 100 μs, two extreme bands disappear (Figure 2b,e). This means that their kinetics is much different (faster) than the one related to the main emission band centered at around 600 or 650 nm for 37 and 39 at.% of Si, respectively. To analyze this aspect further, the same TRPL spectra have been collected in a 100-ns window and recorded just after the 2-ns pulse. From the obtained results shown in Figure 2c,f, it can be seen that only the band on the high-energy side of the main emission can be observed. In this case, the integration window is too small to see the slow, main emission band. This band is related to the levels which just started to be populated. Some indication of this band can be seen as a second emission component shown in Figure 2c. Moreover, the position of defect-related bands is the same for both samples and does not depend on Si content. This is opposite to the behavior of the main band which shifts with Si content towards lower energies. This type of fast short-wavelength emission has been observed already and is considered to be caused most probably by STE. For this band, we were also able to measure the emission decay time, which is equal to 20 ns for both samples. Due to system limitations and weak signal of the main emission band (*a*Si-NCs), we were only able to estimate from TR-PL the average decay time as 500 μs.

Based on the results obtained so far, we conclude that the observed wide emission band obtained usually at CW excitation is a superposition of three emission sub-bands coming from spatially resolved objects with very different kinetics: (1) a band at around 450 nm, with 20-ns decay, which is not changing its position with Si content and is related to optically active defect states and STE in the SRSO matrix; (2) a band at around 600 nm related to *a*Si-NCs with hundreds of microsecond emission decay and strong dependence on Si content following the predictions of the quantum confinement model; (3) and a third band at around 800 nm (1.54 eV) (Si-NCs, defects) with either very fast (<3 ns) or very slow (>100 μs) emission kinetics also depending on Si content. In this case, however, an increase in Si content influences only the relative intensities between this band and the *a*Si-NC emission bands. This band can be related to Si-NCs or defect-related states. The weak dependence of the position of this band on Si content can be due to the weak quantum confinement regime. Based on our previous XRD and Raman results for similar samples, we can assume that the size of Si-NCs is in the range of 4 to 6 nm.

In summary, two components often obtained in emission decay times when the signal is recorded at one energy can be due to different spatially resolved objects (*a*Si-NCs and Si-NCs or defects) rather than two relaxation mechanisms different in timescale related with one object only, i.e., Si-NCs or *a*Si-NCs. The second conclusion that can be given based on the obtained preliminary results is that in many cases, the shift of the emission band at CW excitation observed for samples either annealed at different temperatures or obtained at different excess Si contents can be due to different contributions of defect states into this band. This shift is often related to changes in Si-NC size only. However, at the same time, these two technological parameters change also the number of defects in the matrix, induce a phase transition of Si clusters from amorphous to crystalline, influence the lanthanide distribution [3], and modify the strain at the clusters’ interface, increasing/reducing the tails of density of states [46].

**Figure 3:**
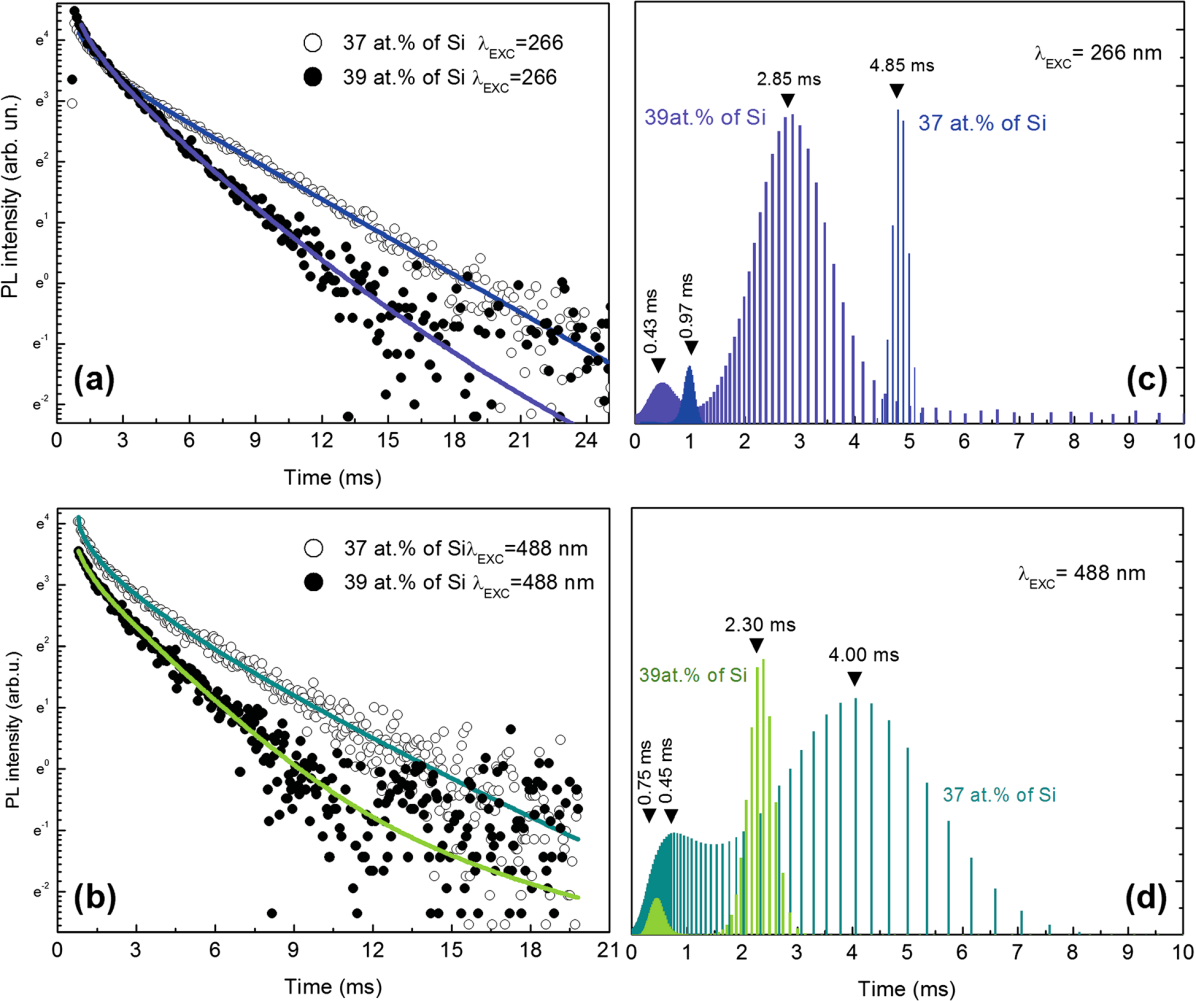
**Time evolution of the 1,535-nm band. **(**a**) PL decay obtained for samples with 37 and 39 at.% of Si at 266 and (**b**) 488 nm. (**c**) MEM distribution of emission decay at 266-nm excitation for 37 and 39 at.% of Si and (**d**) MEM distribution of emission decay at 488-nm excitation for 37 and 39 at.% of Si.

To better understand the dynamics of the Er^3+^-related emission, the time evolution of the 1,535-nm band has been analyzed at different excitation wavelengths: 266 and 488 nm. Figure 3 shows the obtained results together with maximum entropy method (MEM) analysis expressed in the form of *α*(*τ*).

In the analysis of kinetic experiments involving the relaxation of complex materials, such as rare-earth-doped glasses, it is often very difficult to choose appropriate models to fit the data. In particular, it is difficult to distinguish between non-exponential models (such as the ‘stretched exponential’) and models that consist of a few discrete exponentials. Thus, many authors use stretched exponential functions to fit the Er^3+^-related emission decay which, in many cases, is not justifiable. To prove the well-grounded use of two exponential functions to fit our data instead of one exponential or a stretched exponential function, we calculated the inverse Laplace transform of the decay curves obtained by us. This solution allows us to seek a representation for the relaxation process in a space of decay rates, thus obviating the necessity of forcing a particular functional form to fit the data. In this case, the PL decay can be written as

(3) $$I_{\mathrm{PL}}\left(t\right)=\int_{0}^{\infty }g\left(k\right)\text{exp}\left(-\mathrm{kt}\right)\mathrm{dk}$$

where *g*(*k*) is a distribution of decay rate constants for the process *I*(*t*). Given an experimental *I*(*t*), we would like to obtain the appropriate distribution *g*(*k*) that obeys Equation 3, without any assumption about the analytical form of *g*(*k*). This essentially involves performing a numerical inverse Laplace transform of the measured decay *I*(*t*) which can be written as

(4) $$g\left(k\right)=\frac{1}{2\mathrm{\pi i}}\int_{\mathrm{Br}}\text{exp}\left(-\mathrm{kt}\right)I\left(t\right)\mathrm{dt}$$

where the integration is carried out over the appropriate Bromwich contour. The calculation of an inverse Laplace transform on a noisy data function is known from information theory to be an ill-conditioned problem, and a large number of distributions can fit the data equally well. Nevertheless, it is possible to find the distribution *g*(*k*) using the maximum entropy method. The MEM is based on maximizing a function called the Skilling-Jaynes entropy function

(5) $$S=\int_{0}^{\infty }\alpha \left(\tau \right)-m\left(\tau \right)\text{log}\frac{\alpha \left(\tau \right)}{m\left(\tau \right)}\mathrm{d\tau}$$

where *α*(*τ*) is the recovered distribution and *m*(*τ*) is the assumed starting distribution. In this equation, *τ* = 1/*k*, and the relation between *g*(*k*) and *α*(*τ*) is *α*(*τ*) = *τ*^-2^*g*(1/*τ*). MEM allows finding *α*(*τ*) without any previous knowledge that we may have about the rate distribution. This method has been successfully applied in many situations where the inverse problem is highly degenerate, owing to the presence of noise in the data or the large parameter space one is working with.

Thus, based on the above approach, we fit our data with two exponential functions. It should be mentioned that an important aspect of MEM is that even purely exponential decay processes have decay time distributions with finite width (unless the data is completely noiseless). Therefore, the broad distributions obtained by MEM, i.e., in the case of 488-nm excitation for 37 at.% of Si sample, do not necessarily imply non-exponential dynamics. A test to verify this is to fit the data with exponential decays taking the peaks of the distributions as the decay times. In the investigated case, the PL decay can be fitted very well with a two-exponential decay (*χ*^2^ ≈ 1.0), yielding decay times of 4,860 and 885 μs and 2,830 and 360 μs for the samples with 37 and 39 at.% of Si, respectively. The obtained decay times are almost the same as the distribution peaks shown in Figure 3. This result allows us to conclude that the PL decay for both samples can be described by two exponential functions. It should be emphasized that this conclusion could not be drawn without MEM analysis since the PL decays can be fit well also with other models, e.g., the stretched exponential function of the form *I*(*t*) ~ *t*^β-1^∙exp(-(*t*/*τ*)^β^). However, in the case of the stretched exponential function, the distribution *α*(*τ*) should exhibit the power-law asymptotic behavior of the form *α*(*τ*) ~ *t*^β-1^, for *t* → 0, which is not the case.

Thus, at 266-nm excitation for both samples, we obtained emission decay times characterized by two components: a fast one (<1 ms) and a slow one (approximately 3 ms). Since the radiative transitions in Er^3+^ are only weakly allowed, the cross sections for optical excitation and stimulated emission are quite small, typically in the order of 10^-21^ cm^2^. Because of that, the radiative lifetime of the ^4^*I*_13/2_ → ^4^*I*_15/2_ transition in Er^3+^ ions excited directly in SRSO should lie between 14 ms for pure silica [47] and 1 ms for silicon [48].

The longer time obtained by us is typical for times obtained by other authors (i.e., SiO, 2.5 to 3.5 ms [49] and SRSO, 2 to 11 ms [11,50-52]). To explain the second component of our samples, we have three options: (a) Er^3+^ ions are excited via *a*Si/Si-NCs, and there is only one optically active Er^3+^ site excited by two temporally different mechanisms; (b) Er^3+^ ions are excited via *a*Si/Si-NCs, and there are two different Er^3+^ sites, i.e., the isolated ion and clusters of ions; and (c) optically active Er^3+^ ions are excited via Si-NCs and *a*Si-NCs or defect states separately with a different kinetics [53].

Nevertheless, even if the above models could explain two different times recorded for Er^3+^ emission, the short time observed for Er^3+^ seems to be much shorter than expected. This could be explained only by the assumption that the short emission decay can be related to Er^3+^ ions which interact with each other, and due to ion-ion interaction, their emission time can be significantly reduced. Efficient clustering of lanthanides and especially Er^3+^ ions has already been shown by us and other authors [3,25]. Thus, we propose that the slow component is due to emission from isolated ions, while the fast component is related with the ions in a cluster form.

Moreover, from Figure 3, it can be seen that with increase of Si content, the Er^3+^-related emission decay is reduced. We believe that this is due to changes in the refractive index of our matrix for both samples and its contribution to the expression defining the radiative emission time for lanthanides [54]:

(6) $$A_{\mathrm{RJ}\prime J}^{\mathrm{ED}}=\frac{64\pi^{4}e^{2}}{3h{\bar{\lambda }}^{3}\left(2J\prime +1\right)}\frac{n{\left(n^{2}+2\right)}^{2}}{9}{\sum_{\lambda =2,4,6}\Omega_{\lambda }\left|〈\mathrm{\Psi J}\left|U^{\lambda }\right|\Psi \prime J\prime 〉\right|}^{2}$$

(7) $$\frac{1}{\tau_{R}}=\sum_{J}A_{R\left(J\prime J\right)}^{\mathrm{ED}}$$

where *n* is the refractive index of the matrix, <*ΨJ*′| and |*ΨJ*> are the initial and final states of single parity, *U*^(λ)^ is the irreducible tensor form of the dipole operator, *λ* is the emission wavelength, and Ω_λ_ are the Judd-Ofelt parameters, describing the local environment of the ion. We have observed similar effects of the influence of *n* on the emission decay time recently for Tb^3+^ ions introduced into a SRSO matrix where the Si concentration was changed from 35% to 40%, increasing the refractive index from 1.55 to 1.70. Additionally, this reduction in decay time can be also due to an increased number of non-radiative channels with increasing Si content making contributions to the final emission decay as *τ*_PL_^-1^ = *τ*_R_^-1^ + *τ*_NR_^-1^. Similar results have been obtained when 488 nm was used as the excitation wavelength. Moreover, reduction in emission decay time has been observed when the excitation wavelength is changed. The emission decay time at 488 and 266 nm can be different when two different sites are excited at different wavelengths. To verify this, emission spectra of Er^3+^ ions obtained at 10 K have been compared at different excitation wavelengths. In the limit of our system resolution, we did not find any difference in the emission peak position at different excitation wavelengths. Thus, we believe that the same sites emit at 1,535 nm at all excitation wavelengths.

### Thermal quenching

To investigate the effect of emission quenching, we have performed PL measurements as a function of temperature for different excitation wavelengths. In order to interpret these results, we considered the temperature dependence of the PL intensity at low pump power according to the Arrhenius law with *E*_Q_ as deactivation (ionization) energy.

Based on the FTIR and Raman spectroscopy done on our samples previously [46], we found several absorption bands related with phonons or SRSO matrix vibrations which can participate in thermal quenching. Typical Raman spectra obtained by us for these samples consist of two bands: a broad low-frequency band (LF) with maximum at around 485 cm^-1^ (59 meV) and a narrower, asymmetrically broadened high-frequency (HF) peak centered at 520 cm^-1^(64 meV). The LF band may be attributed to *a*Si present in the matrix, whereas the HF originates from Si-NCs. Moreover, from the FTIR spectra, there are three main bands located at 1,000 to 1,300 cm^-1^ (123 to 161 meV) and 800 cm^-1^ (100 meV) related to the asymmetric stretching and bending Si-O-Si modes, respectively.

In general, the quenching of the luminescence with temperature can be explained by thermal emission of the carriers out of a confining potential with an activation energy correlated with the depth of the confining potential. Since the observed activation energy is much less than the band offsets between Si/SiO_2_ (approximately 3.4 eV), the thermal quenching of the *a*Si/Si-NC-related emission is not due to the simple thermal activation of electrons and/or holes from the *a*Si/Si-NCs potential into the SiO_2_ barriers. Instead, the dominant mechanism leading to the quenching of the VIS-related PL is due to the phonon-assisted tunneling [55] of confined carriers to states at the interface between *a*Si/Si-NCs and the matrix.

**Figure 4:**
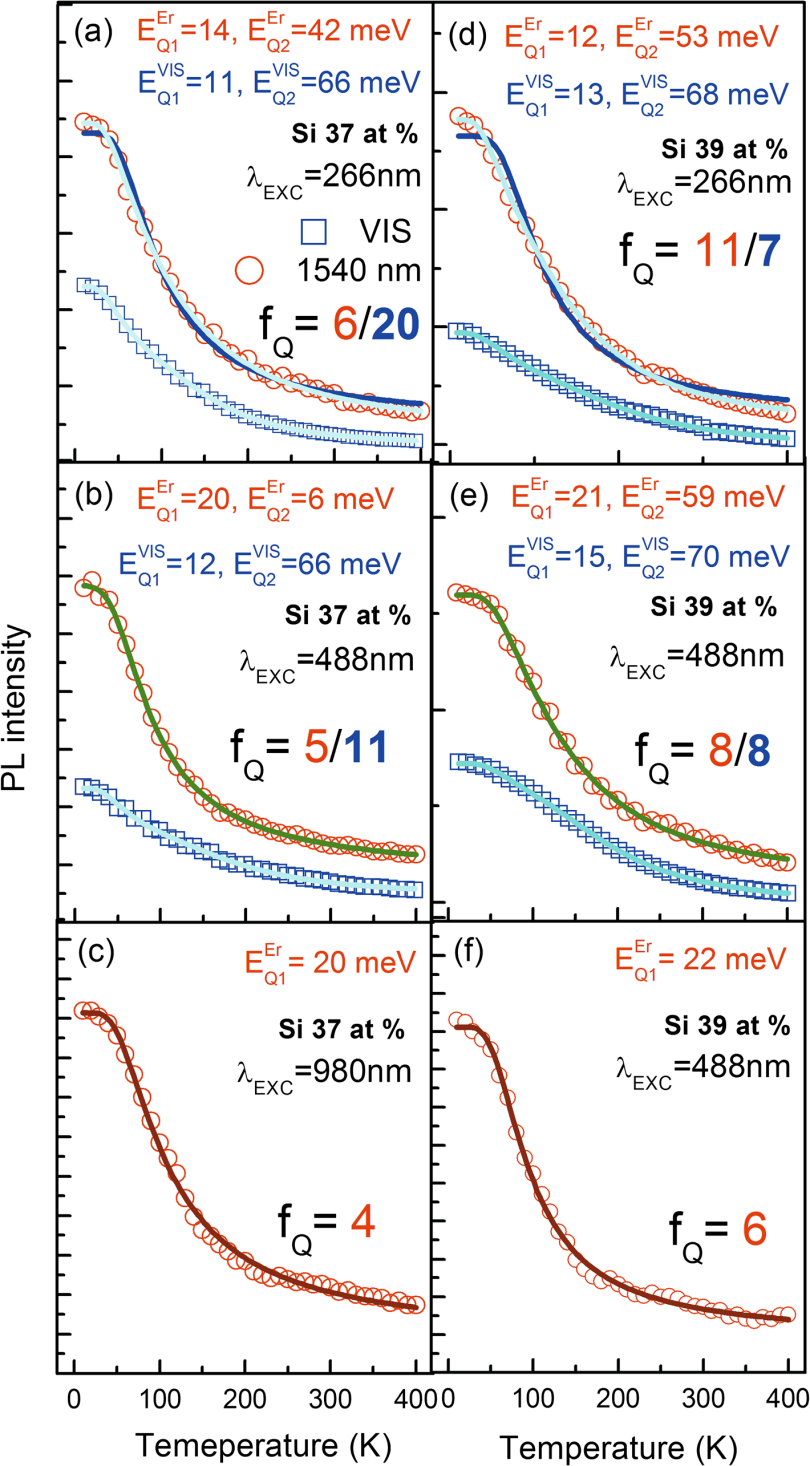
**Emission thermal quenching. **Obtained for Si-NCs and Er^3+^-related bands at different excitation wavelengths (266, 488, and 980 nm) as function of temperature for two samples with 37 (**a, b, c**) and 39.at % of Si (**d, e, f**). Photon flux used for the experiment was equal to: Φ_266 nm _= 8 × 10^19^, Φ_488 nm _= 56 × 10^19^, Φ_980 nm_ = 570 × 10^19 ^(photons/s × cm^2^) for 266, 488, and 980 nm, respectively. These fluxes correspond to the lowest excitation power allowing performance of the experiment and are equal to excitation power of 0.6, 6, and 40 mW for 266, 488, and 980 nm, respectively. Abbreviations used are as follows: *f*_Q_, relative change in emission intensity at 10 and 500 K; *E*_Q_, quenching energy from Arrhenius fit.

As it can be seen from Figure 4c,f, for the excitation wavelength of 980 nm, thermal quenching of Er^3+^-related emission for both samples can be well characterized with only one deactivation energy (*E*^Er^_Q1_) equal to approximately 20 meV. Since the *f* levels of Er^3+^ ions weakly couple to any matrix states due to screening effects of electrons filling higher orbitals, we believe that the observed quenching energy can be related with two mechanisms: Boltzmann distribution of carriers among the Stark levels having different radiative and non-radiative decay probabilities with one multiplet, or phonon-assisted dipole-dipole coupling between the ^4^*I*_13/2_ → ^4^*I*_15/2_ transition and energy levels related with *a*Si/Si-NCs or defect states. The matrix-related emission in this spectral range with nanosecond dynamics has been shown already by other authors [16,56], making this process highly probable. Moreover, we can see that the intensity of the Er^3+^-related emission at this excitation varies by factors of 4 and 6 for samples with 37 and 39 at.% of Si. This is quite a significant change for RE^3+^, suggesting that the main quenching is due to the coupling of Er^3+^ ions with some defect states. We can also see that this quenching is almost twice as large for the sample with 39 at.% of Si, suggesting correlation of these quenching centers with Si content in the SRSO matrix.

Analyzing the data presented in Figure 4a,d, we can see that when the Er^3+^ is excited with 266 nm, PL thermal quenching can be well fitted only when two quenching energies are used. For both samples, these energies are equal to *E*^Er^_Q1_ ~ 15 meV and E^Er^_Q2_ ~ 50 meV. For comparison, in Figure 4a,d, two fits have been shown with one and two quenching energies. It is clear that two energies are needed to obtain a statistically good fit. Once we look at thermal quenching recorded for the emission related to *a*Si/Si-NCs, we can see that the thermal quenching can also be fitted with two energies similar for both samples: *E*^VIS^_Q1_ ~ 10 and *E*^VIS^_Q2_ ~ 65 meV.

The *E*^VIS^_Q2_ energy corresponds exactly to the energy of phonons related to oscillations of Si-Si bonds obtained in Raman experiments. In more detail, this value is closer to the amorphous phase of silicon rather than the crystalline phase. This could be related to the fact that amorphous nanoclusters are responsible for the observed emission in the VIS range as well as for the indirect excitation of Er^3+^ ions. Thus, most probably at a temperature corresponding to 65 meV, one of the carriers is moved from the potential related with *a*Si-NCs to defects states at their surface, where it recombines non-radiatively or diffuses over longer distances inside the matrix. The second energy (*E*^VIS^_Q1_) is much less clear at the moment. Nevertheless, correlation between the second quenching energy (55 meV) observed for Er^3+^ emission with the quenching energy obtained for *a*Si-NC emission (65 meV) suggests efficient coupling between these two objects and confirms that most of the quenching appears before the excitation energy is transferred from *a*Si-NCs to Er^3+^ ions. Also, at this excitation, Er^3+^-related emission is quenched by factors of 6 and 11 for samples with 37 and 39 at.% of Si, respectively.

Figure 4e shows results of thermal emission quenching at 488-nm excitation wavelength for a sample with 39 at.% of Si. It can be seen that the Er^3+^-related emission is also characterized by two quenching energies equal to about 20 and 60 meV. These values are almost the same as for 266-nm excitation and very similar to VIS emission where values of 15 and 70 meV have been obtained. This indicates that in this case also, we deal with indirect excitation of Er^3+^ ions. Since 488 nm corresponds also to direct excitation of Er^3+^ ions, most probably, we deal with both kinds of excitation simultaneously. We believe, however, that indirect excitation is in this case dominant. Nevertheless, the results obtained at this excitation wavelength for 37 at.% of Si are not so obvious. In this case, two statistically equal fits with one (20 meV) and two energies (20 and 6 meV) were possible to achieve. The higher energy is clear and has the same origin as in the previous cases. One explanation of this fact would be the excitation spectrum for this sample where its edge is much shifted to blue as compared to samples with 39 at.% of Si. Thus, in this case, we can indeed observe a major contribution from a direct excitation of Er^3+^ ions rather than via intermediate states.

## Conclusions

The existence of efficient excitation transfer from silicon nanoclusters to Er^3+^ ions has been shown for SRSO thin films deposited by ECR-PECVD by means of PL, TRPL, PLE and temperature-dependent PL experiments. However, it has been shown that for our samples, this energy transfer is most efficient at high excitation energies. Much less efficient energy transfer has been observed at 488-nm excitation. In this case, depending on Si nanocluster size, we deal with dominant contribution to Er^3+^ excitation from indirect excitation channel (big nanoclusters) or from direct excitation of Er^3+^ ions (small nanoclusters).

Moreover, it has been shown that a wide emission band in the VIS spectral range is a superposition of three emission sub-bands coming from spatially resolved objects with very different kinetics: a band at around 450 nm, with 20-ns decay, which is not changing with Si content and is related with optically active defect states and STE in SRSO matrix; a band at approximately 600 nm related to *a*Si-NCs with hundred-microsecond emission decay and strong dependence on Si content following the predictions of quantum confinement model; and a third band at around 800 nm (1.54 eV) (Si-NCs, defects) with either very fast (<3 ns) or very slow (>100 μs) emission kinetics, also depending on Si content. Additionally, it has been shown that two Er^3+^ sites are present in our samples: isolated ions and clustered ions with emission decay times of approximately 3 and <1 ms, respectively.

## Competing interests

The authors declare that they have no competing interests.

## Authors’ contributions

AP, GZ, LG, and JM carried out the spectroscopic measurements. JW and PM designed and deposited the investigated samples. All authors read and approved the final manuscript.
